# Correction: TFAP2 paralogs facilitate chromatin access for MITF at pigmentation and cell proliferation genes

**DOI:** 10.1371/journal.pgen.1010378

**Published:** 2022-08-29

**Authors:** The PLOS Genetics Staff

Fig 6 is incorrect. Magnified images do not appear in the figure. The publisher apologizes for the error. The authors have provided a corrected version of Fig 6 here.

**Fig 6:**
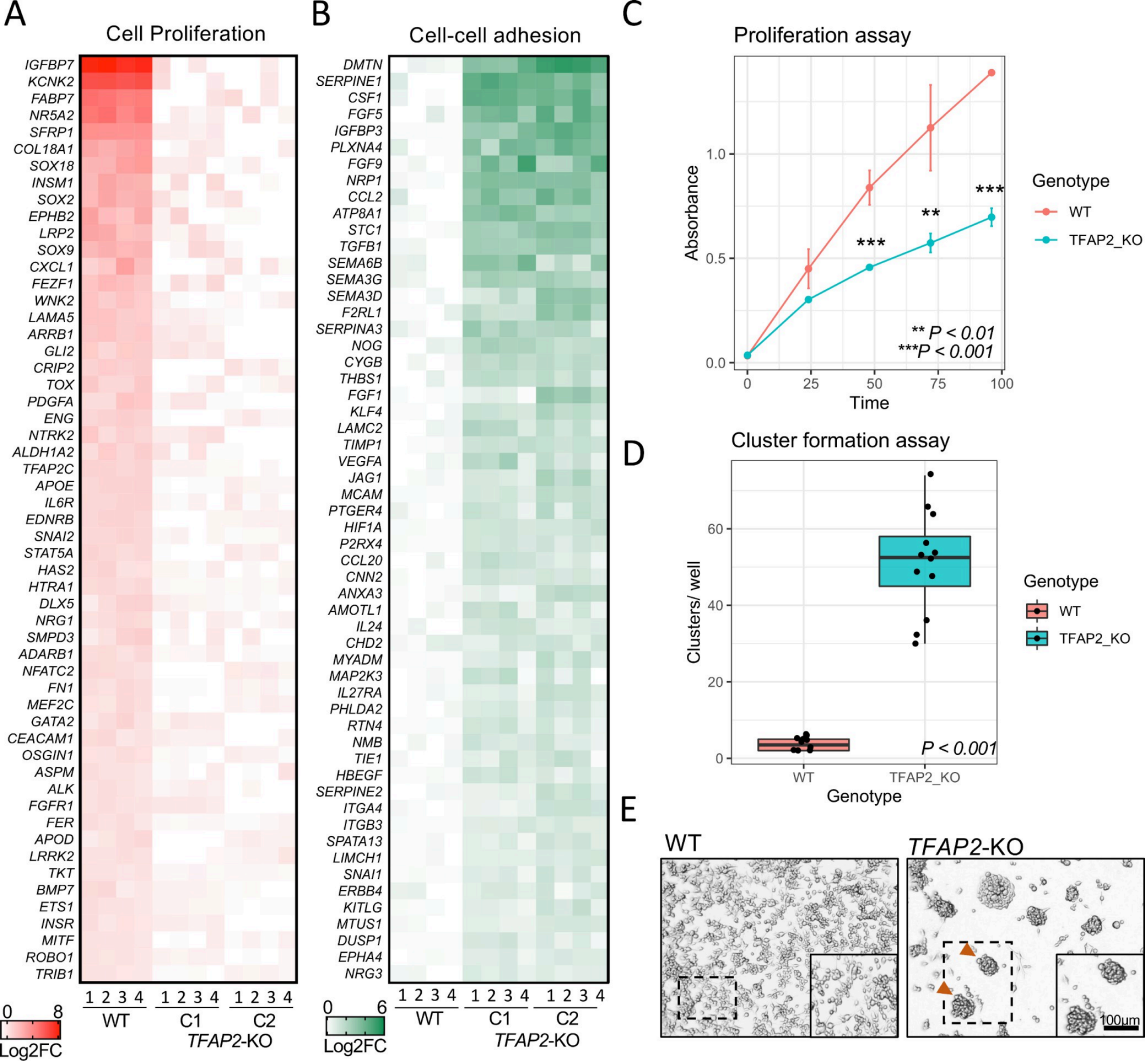
TFAP2 paralogs promote cell proliferation and inhibit cell-cell adhesion a melanoma cell line. (**A**) Heatmap representing the top 55 directly TFAP2-activated genes and (**B**) the top 55 directly TFAP2-inhibited genes that are associated with the GO terms cell pigmentation and cell-cell adhesion, respectively. Four replicate RNA-Seq experiments are shown for WT cells and two clones of *TFAP2*-KO cells (Clone 2.12 and Clone 4.3) (FDR < 0.05). (**C**) Growth curves (mean ± SE of mean) over 100 hours of cultivation for WT and *TFAP2*-KO SK-MEL-28 cells. x-axis is time and y-axis is absorbance at 450nm which is directly proportional to number of living. (**D**) Box plots representing the quantification of cluster formation on low-bind plates after 72 hours of culture (n = 12 independent experiments, p < 0.001 by Student’s t-test, plot shows mean ± SD). (**E**) Representative images of clusters formed in WT and *TFAP2*-KO cells after 72 hours.
