# Supplementary figures and images for: TFAP2 paralogs facilitate chromatin access for MITF at pigmentation and cell proliferation genes
Source: PLoS Genet. 2022 May 17;18(5):e1010207. doi: 10.1371/journal.pgen.1010207 (PMC9159589; doi:10.1371/journal.pgen.1010207)

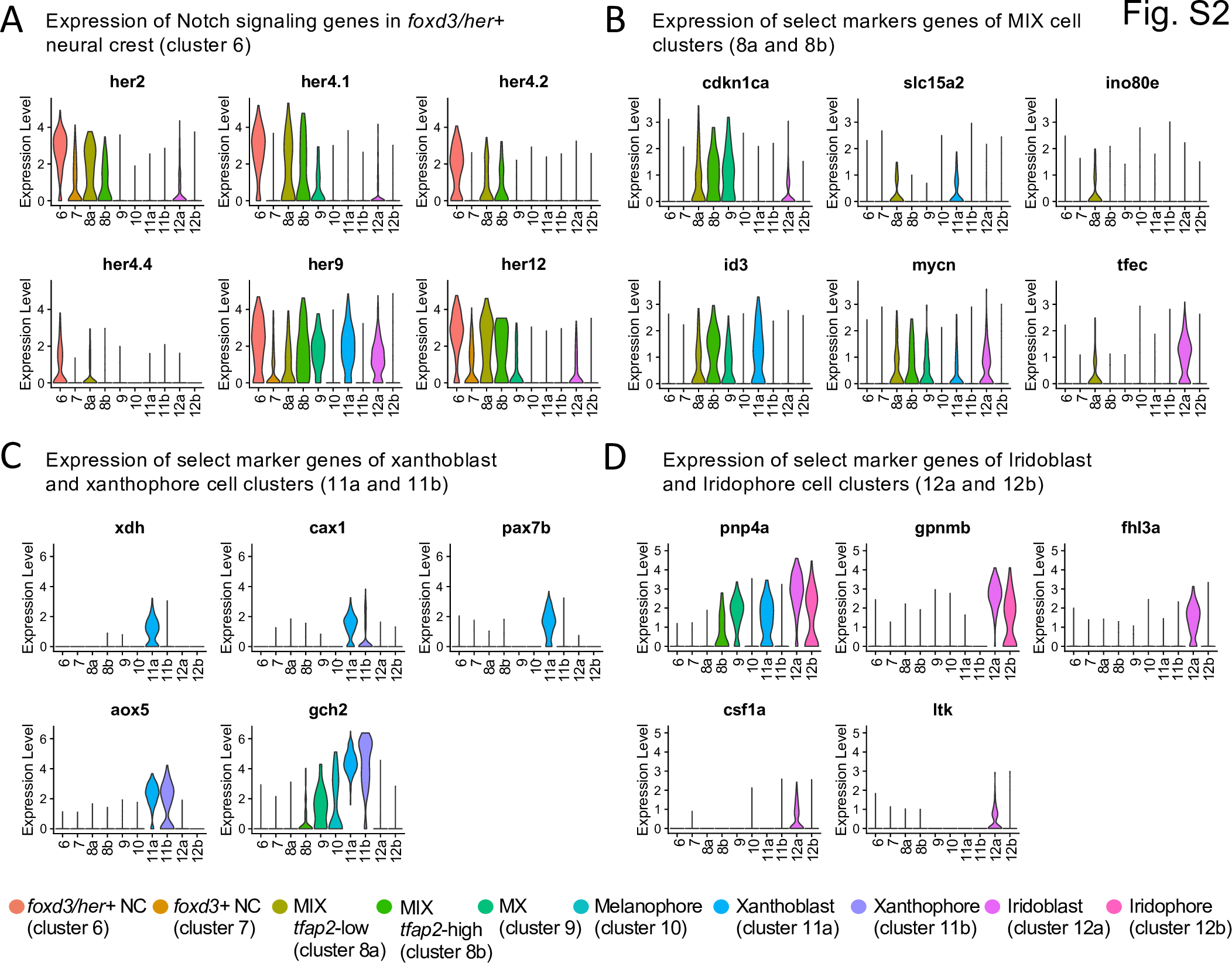

Supplement: S2 Fig — (A) Violin plots showing expression of Notch pathway genes in foxd3/her+ NC cluster 6. Note much lower of Notch signaling gene expression in foxd3+ NC cluster 7. (B) Violin plots showing expression of select genes in MIX cell clusters, some of which are also expressed in MX, xanthoblast and iridoblast cell clusters. (C) Violin plots showing expression of select marker genes in xanthoblast and xanthophore cell clusters. (D) Violin plots showing expression of select marker genes in iridoblast and iridophore cell clusters. (TIF) [file pgen.1010207.s002.tif]

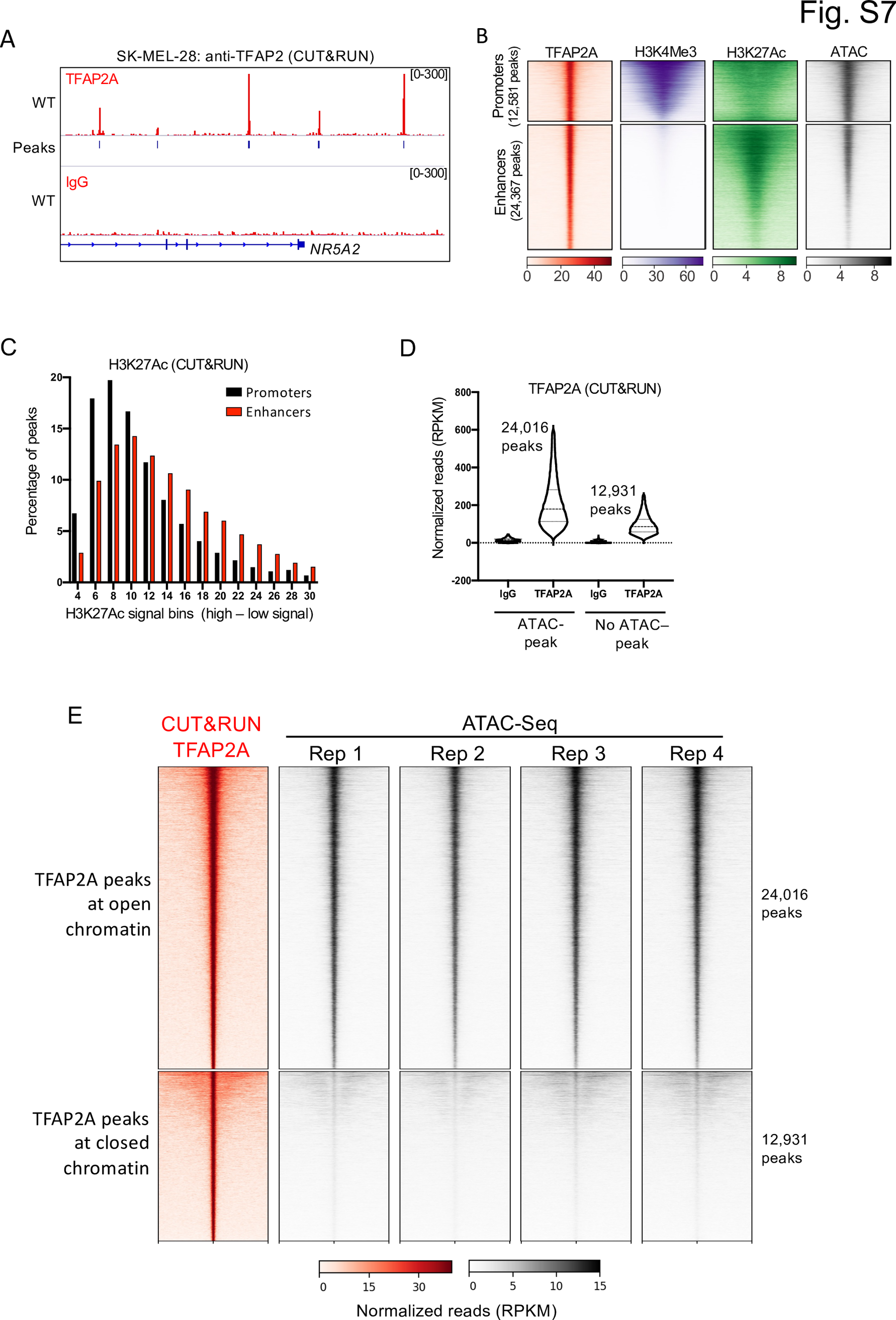

Supplement: S7 Fig — (A) Screenshot of IGV genome browser (GRCH37/hg19), visualizing anti-TFAP2A and IgG CUT&RUN-seq profiles. Peaks were called using MACS2 software (two independent replicates) and are illustrated by blue bars under the anti-TFAP2A track. (B) Density heatmap centred on the 36,867 TFAP2A peaks identified by anti-TFAP2A CUT&RUN in WT SK-MEL-28 cells. Regions shown are +/- 3 kb from peak center. Promoter peaks are < 3kb and enhancer peaks >3kb from a transcriptional start site (TSS). Anti-TFAP2A, anti-H3K4Me3 and anti-H3K27Ac CUT&RUN-seq, and ATAC-Seq profiles are shown. Color code reflects normalized Reads Per Kilobase, per Million mapped reads (RPKM). (C) Histogram representing H3K27Ac signal, binned from low to high read-depth (normalized RPKM) on the x-axis and percentage of TFAP2A promoter peaks (black) and TFAP2A enhancer peaks (red) on the y-axis. (D) Violin plots illustrating TFAP2A and IgG normalized reads (RPKM) at open (ATAC-peaks) and at closed chromatin (no ATAC-peak) (E) Density heatmap representing TFAP2A CUT&RUN and ATAC-seq profiles at TFAP2A peaks that overlap nucleosome depleted regions (ATAC-peaks) and at nucleosome bound DNA (no ATAC-peak), the number of TFAP2A peaks in each group are as labeled. (TIF) [file pgen.1010207.s007.tif]

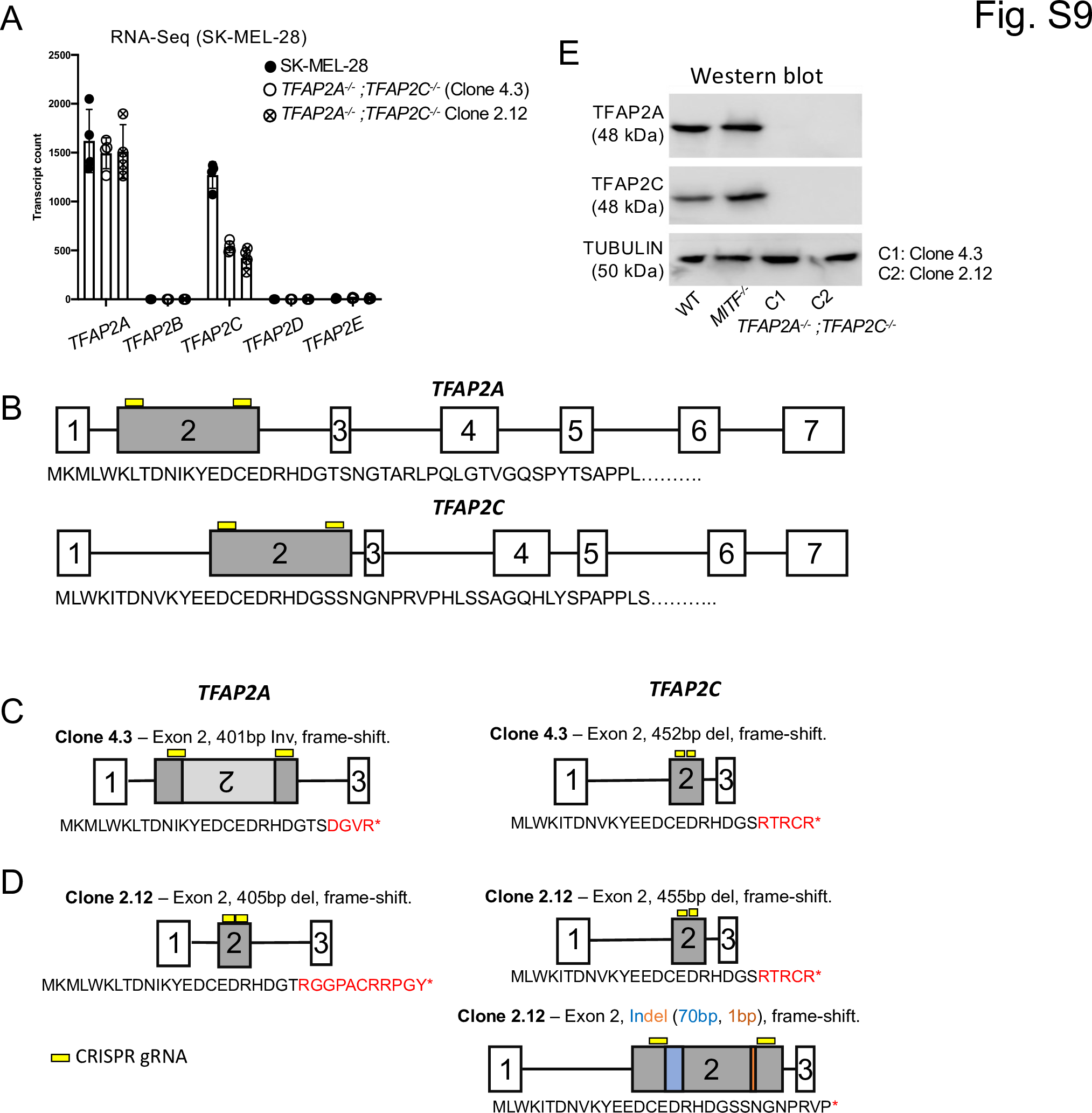

Supplement: S9 Fig — (A) RNA-seq showing transcript counts of TFAP2 paralogs in SK-MEL-28 cells. Transcript counts for WT cells (n = 4) and two TFAP2A;TFAP2C double knockout clones (4 replicates each) are shown. The expression of WT and mutant alleles of TFAP2A is comparable between cell lines whereas mutant alleles of TFAP2C are strongly reduced. (B) Two CRISPR guide RNAs (gRNA) each were designed to target exon 2 of TFAP2A and TFAP2C (yellow boxes). (C) A 401 base pair inversion and a 452 base pair deletion at exon 2 of TFAP2A and TFAP2C, respectively, was identified in clone 4.3. TFAP2A and TFAP2C mutant alleles resulted in a frame-shift and premature stop codon in alleles of both genes. (D) A 405 base pair deletion at exon 2 of TFAP2A resulted in a frame-shift and premature stop codon in clone 2.12. A 455 base pair deletion, and a 70 base pair insertion, 1 base pair deletion (Indel) was identified in exon 2 of TFAP2C. Such mutations resulted in a frame-shift and premature stop codon. Additional permutations were not identified at exon 2 of TFAP2A or TFAP2C in clone 4.3 or clone 2.12 cells. Inv, inversion; Del, deletion. (E) Western blot analysis confirming loss of TFAP2A and TFAP2C immunoactivity in clone 4.3 and clone 2.12 cell lines. (TIF) [file pgen.1010207.s009.tif]

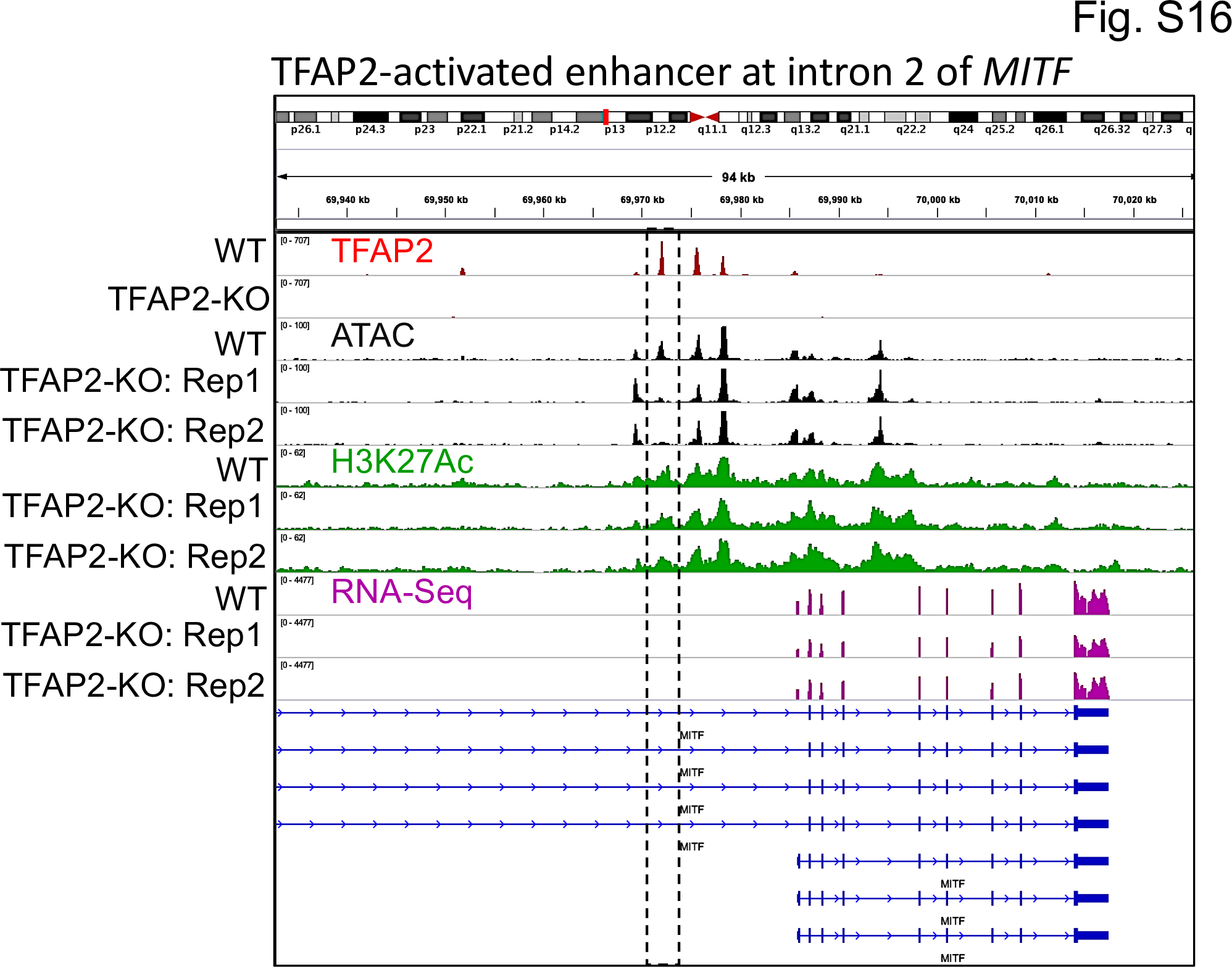

Supplement: S16 Fig — Screenshot of IGV genome browser (GRCH37/hg19) visualizing anti-TFAP2A, ATAC-seq, anti-H3K27Ac and RNA-seq profiles at intron 2 of MITF. Dashed rectangle indicates an TFAP2-activated ATAC-seq peak; like the ATAC-seq signal, the H3K27Ac-signal is significantly lower in TFAP2-KO cells relative to in WT cells implying this is a TFAP2-pioneered-and-activated enhancer, although the log2 fold change of the H3K27Ac signal does not meet the cut-off we used to define this category of enhancer elsewhere in the paper. MITF and downstream regions are shown, blue arrows indicate strand orientation and horizontal rectangles the exons. Genotypes are as labeled; y-axes are grouped scaled per dataset. (TIF) [file pgen.1010207.s016.tif]
